# A Case of Bronchitis and Pneumonia Caused by the Inhalation of the Filling from a Tooth Broken in Extraction

**Published:** 1899-07

**Authors:** Charles O'Donovan

**Affiliations:** Baltimore, Md.


ARTICLE III.

### A CASE OF BRONCHITIS AND PNEUMONIA CAUSED BY THE INHALATION OF THE FILLING FROM A TOOTH BROKEN IN EXTRACTION.

CHARLES O'DONOVAN, M. D., BALTIMORE, MD.

On March 21, 1891, I was asked to see Mrs. F. B., White, aged forty-six, married, mother of three children, the oldest about twenty-three. She is a stout woman, of medium height, with very florid complexion, who had always been healthy except for occasional colds that were hard to shake off, and often caused mild bronchitis. On the day before she had had a tooth extracted, under the influence of nitrous oxide, by a perfectly competent dentist, but he had broken the tooth and had caused her a considerable amount of pain in the operation. She took the gas but once, but did not recover well from its inhalation. She felt very much "stuffed up" after returning home, and breathed with difficulty. She thought that she had taken cold while at the dentist's, but paid little attention to the matter, fully expecting to be rid of it by the next morning. On the contrary, she was much worse; she had a very restless night, sleeping but little on account of the oppressed breathing, and being distressed by a teasing but uncontrollable cough, which was rasping and dry, coming on in paroxysms, but giving rise to no expectoration. I examined her throat and chest very carefully, but could detect nothing wrong; so I ordered a simple expectorant, containining a slight amount of morphine, and told her that she would soon be all right. By the next day I had reason to change this opinion, for I found her in the same condition, having coughed nearly all night, her nervous system rapidly showing signs of distress, her pulse up to 119, and temperature to 101°, and a process of bronchitis developing in the medium-sized bronchi about two-thirds down the right lung and midway between the spine and the axillary line. From this the bronchitis spread in a few days throughout the entire lower third of the right lung, with considerable elevation of temperature and quickened pulse rate, accompanied by great prostration. At the same time her expectoration became quite free, beginning as mucus and froth, but rapidly becoming purulent. Meanwhile the other lung remained perfectly clear throughout. In spite of absolute rest in bed, the best of nourishment, excellent nursing, and various different expectorations and sedatives, she continued to grow worse daily; she lost her appetite, she slept very irregularly and without refreshment, her bowels became costive, her cough continued, she complained always of an oppression about her chest, and began to have a cutting pain in the right lung with each cough or forced inspiration. On April 10th a distinct dull spot was made out just where the original focus of inflammation had been; it was not pneumonic, it had no tendency to spread, but seemed to be a dead, flat spot about as large as a pigeon's egg, surrounded by an area of dull, infiltrated tissue, that faded imperceptibly into the bronchitis surrounding the area. This spot grew slightly larger as time passed, but no new foci developed anywhere else; the upper two-thirds of the lung remained clear and resonant, but the lower third was very dull upon percussion, almost approaching a hypostatic pneumonia, with moist rales in the medium and finer tubes to be heard all through the inflamed area. The left lung showed also some few moist rales, but no dullness. She had every evening a rise of temperature to 102° to 104° F., with a fall during the morning to 99° to 100° F. She was evidently very ill, but unaccountably so. Tuberculosis was thought of, as her family history showed some taint, but the sudden onset and anomalous development of the trouble made it unlikely; however, some of the sputa was examined and showed no bacilli. Her nourishment was forced, and she was encouraged to sit up a while each day, but she was not improved, and asked to be allowed to stay in bed. Quinine and other antipyretics were given, but had no effect upon the fever, which became more and more hectic in character, with chilly sensations before the rise, and the expectoration began to have blood in it in increasing quantities, never any large amount, but quite as much as in pneumonia. By the end of April the original spot showed evidence of softening, and the formation of a cavity soon followed: it was clearly defined, not larger than a walnut, with well-marked amphoric breathing, some ægophony, but very little or no bubbling. In the rest of the lung there was no change in the general condition, the area of inflammation and infiltration did not extend, nor did the bronchitis show any sign of improvement. After consultation the condition was readily agreed upon, but no reason could be assigned for this trouble, nor could a satisfactory prognosis be given. Her cough continued to be always troublesome, interfering much with her rest at night, and giving rise to a very free expectoration of thick yellow stuff, with frequent admixture of a little blood; her pulse was rather hard and jerky, beating about one hundred to the minute, and her temperature was constantly above the normal, with a suspiciously regular rise each afternoon. Except for the inability to find bacilli, and her excellent health in the past, I should have diagnosticated tuberculosis without hesitation. All through May and early June she continued in this condition. She had a trained nurse, who devoted great care to her nourishment, that being given the first place in treatment rather than any medicines; of these, various expectorants and tonics were used, including a fair allowance of whisky. As a result she lost no flesh, rather gaining than otherwise, and she presented no appearance of illness beyond the frequent cough with expectoration. Her symptoms, however, pointed to a very serious condition. She described herself as constantly breathing with the greatest oppression, and so weak that the slightest exertion caused her to become faint and brought on severe paroxysms of coughing, although she was forced to get up every day in spite of daily protests. The lowest third of her right lung became gradually waterlogged, with general, diffuse bronchitis through it, giving rise to moist rales everywhere, with exudation and infiltration, especially as the original focus was approached, this being a cavity surrounded by a zone of consolidation. In the left lung some bronchitis appeared also, but this affected only the medium-sized tubes and did not produce much extra-bronchial infiltration. On June 17th she went to Atlantic City, being in the same general condition, with oppressed breathing, frequent cough, much purulent and some little bloody expectoration, no appetite, but taking food under compulsion, sleeping badly, and in a very wretched frame of mind. It was hoped that the sea air would benefit her, but she failed to respond in any way to the change. On July 7th—more than fifteen weeks from the time that she had so suddenly and peculiarly developed her bronchitis—in a severe paroxysm of coughing she spat up, with the usual purulent and bloody mucus, a piece of amalgam filling from the broken tooth, smooth on one surface, but very rough and jagged on the other, where it had been joined to the decayed and excavated remnant of the tooth; it formed approximately a parallelogram measuring half an inch long, three-eighths of an inch wide, and having a thickness of an eighth of an inch; it weighed 28.11 grains. Although very doubtful of the final outcome of such prolonged irritation, I wrote most hopefully to her, and advised her to stay on at Atlantic City. At first her improvement was very slow, causing her much discouragement, and leading her to leave the seashore early in August for a Blue Ridge Mountain resort; here she grew gradually stronger and better, gaining in various ways. I did not see her until late in September, when I found her practically well, except for the cavity in the right lung, evidently the place where the foreign body had been lodged. This cavity, although perfectly apparent and readily made out, was much smaller than it had been in June, and slowly contracted during the succeeding months, disappearing finally during the winter. She continued, for several years, more than usually susceptible to colds, and always found with each new cold that bronchitis was apt to develop in the right lung with a good deal of pain at the side of the cavity. In time this disappeared also, and she is now (October, 1898) perfectly well.—*New York Medical Journal,* Nov. 26th, 1898.

---

ARTICLE IV.

